# Still Bay Point-Production Strategies at Hollow Rock Shelter and Umhlatuzana Rock Shelter and Knowledge-Transfer Systems in Southern Africa at about 80-70 Thousand Years Ago

**DOI:** 10.1371/journal.pone.0168012

**Published:** 2016-12-12

**Authors:** Anders Högberg, Marlize Lombard

**Affiliations:** 1 Linnaeus University, Faculty of Art and Humanities, Department of Cultural Sciences, Archaeology, Kalmar, Sweden; 2 Department of Anthropology and Development Studies, University of Johannesburg, Auckland Park, South Africa; 3 Stellenbosch Institute for Advanced Study (STIAS), Wallenberg Research Centre at Stellenbosch University, Stellenbosch, South Africa; Universidade do Algarve, PORTUGAL

## Abstract

It has been suggested that technological variations associated with Still Bay assemblages of southern Africa have not been addressed adequately. Here we present a study developed to explore regional and temporal variations in Still Bay point-production strategies. We applied our approach in a regional context to compare the Still Bay point assemblages from Hollow Rock Shelter (Western Cape) and Umhlatuzana Rock Shelter (KwaZulu-Natal). Our interpretation of the point-production strategies implies inter-regional point-production conventions, but also highlights variability and intra-regional knapping strategies used for the production of Still Bay points. These strategies probably reflect flexibility in the organisation of knowledge-transfer systems at work during the later stages of the Middle Stone Age between about 80 ka and 70 ka in South Africa.

## Introduction

The Still Bay phase or technocomplex occurs in southern Africa during the later stages of the Middle Stone Age at roughly 80–70 ka (for summary of the complete South African Stone Age technocomplex sequence as represented by dated sites see Lombard et al. [1], and Henshilwood [2] for historical synthesis regarding the Still Bay). The archaeological record from this time is considered to contain multiple lines of evidence that indicate enhanced cognitive and behavioural trends that are comparable to those of humans today (for most recent synthesis see Wadley [3]). The unique way in which humans are able to process information makes us behaviourally more flexible than any other organism [4]. Our extraordinary flexible nature is expressed in, amongst other things, variations in the production and use of our technologies [5]. Such suppleness provides the ability to adapt effectively to new conditions or situations (personally, socially, economically and ecologically). The associated behavioural flexibility applies to long-term adaptability and change, as well as to instantaneous decision-making processes. Our highly developed behavioural and cognitive flexibility facilitates teaching and learning (the transfer of knowledge across groups or generations), technological innovation and plastic responses to new or complex situations [5–9].

In their integrative macro-scale approach to understanding the Middle Stone Age of southern Africa, Kandel and colleagues [10] conclude that central to this period is ‘its overall variability’, and that behavioural flexibility can be considered to have become the main adaptive driver (also see Shea [11] on behavioural variability *vs* modernity). Variation in lithic tool production has been stressed as key to understanding behavioural flexibility within the later stages of the Middle Stone Age of southern Africa [3], [12–25]. Soriano and colleagues [23], however, note that questions of technological traits and patterning in Still Bay lithic industries have hitherto been dealt with inadequately, and Archer and colleagues [22] conclude that not enough consideration has been given to ‘diachronic and synchronic’ trends within the Still Bay technocomplex.

Although we agree with them in principle, it is our opinion that despite much effort, dating resolution across the Still Bay technocomplex is currently too sparse and problematic to attempt variation through time on a regional scale. Thus far, all published age estimates for the Still Bay in southern Africa range roughly between 80 ka and 70 ka, with the exception of a single site for which similar and older ages have been calculated [1], [25–32]. Until finer dating resolution becomes available for more sites, we are parsimonious by starting a regional exploration of variation in Still Bay point production that might reflect techno-behavioural knowledge-transfer systems between about 80 ka and 70 ka in the southern African Middle Stone Age.

The transfer of knowledge can be defined as a process through which one social unit (an individual, group or community), is impacted on by the experience of another unit [33]. With knowledge-transfer systems we here refer to processes of inter- and intra-generational social learning and how teaching and learning is organised in societies [9], [34]. d’Errico and Banks [6] emphasised various dimensions–spatial, temporal and social–involved in such transfer systems. Most recently, Charbonneau [35] reinforced how, in addition to the informational aspect of social transmission (i.e., the learning, stabilising, and transformation of mental representations along cultural lineages), such systems are governed by the production of public displays, for example, utterances, behaviours, and artefacts. “[T]hese displays are what social learners learn from” ([35], page 1). He also highlights how the ‘generative processes’ that are associated with the production of public displays most often become abstracted and hidden during constructions of theoretical assessments and formal models. This echoes Janette Deacon’s observation that:

Metric and numerical analyses have served their purpose in placing limits on the range of variability to be expected and in enabling inter-site comparisons to be made. By focussing on the tools instead of the toolmakers, however, the humanity and creativity of the people who made the artefacts has been given less attention, making it seem as if they were the victims rather than the perpetrators of change ([36], page 58).

For the purposes of this paper, and in line with what Deacon [36] suggested more than 25 years ago, we focus on toolmaker performances by comparing point-production strategies. Our aim is to refine understanding of different knapping strategies in Still Bay point production, based on a new analysis of artefacts from two Still Bay levels of the Umhlatuzana Rock Shelter assemblage, which we compare to an updated analysis of the previously published results from Hollow Rock Shelter [25]. Based on our outcomes, we propose a blended techno-behavioural knowledge-transfer system. This hypothesis can be tested by future research in relation to, not only spatial, but also temporal and social dimensions of knowledge-transfer systems in Middle Stone Age assemblages [23], [37], [38].

## Hollow Rock Shelter and Umhlatuzana Rock Shelter

Both Hollow Rock Shelter and Umhlatuzana Rock Shelter are well-published sites. We therefore only introduce them briefly here to contextualise our study (for full site descriptions, more extensive records on dating, raw material use, detailed lithic and other data see the primary publications [24], [25], [27], [39–47]).

**Fig 1:**
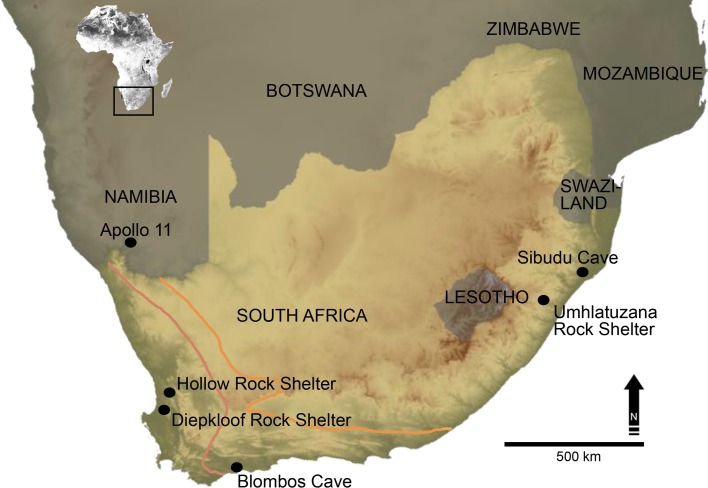
(adapted from Lombard et al. [27]). Map showing the location of Still Bay sites mentioned in our text. Current rainfall zones are marked with pink and yellow lines. East of the yellow line is the summer rainfall zone. West of pink line is the winter rainfall zone. Between the lines is the year-round rainfall zone.

**Fig 2:**
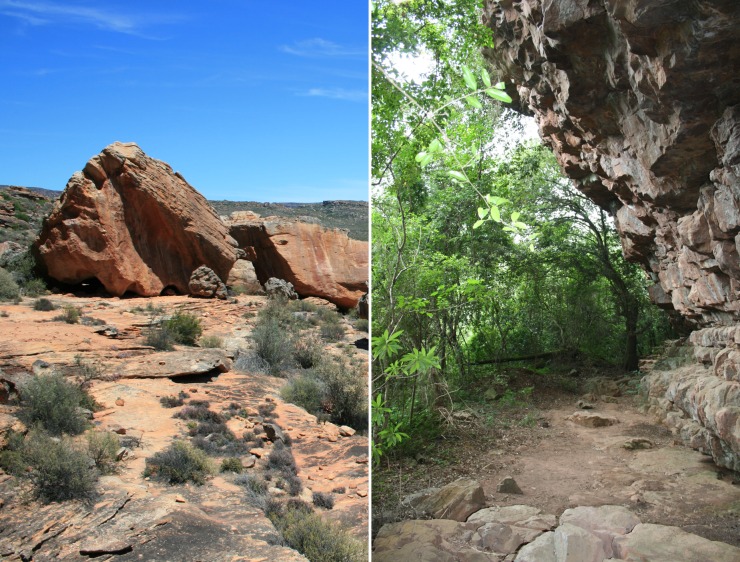
Outside Hollow Rock Shelter Site (left), and inside Umhlatuzana Rock Shelter (right).

Hollow Rock Shelter is located in the northern part of the Cederberg Mountains of the Western Cape Province, which currently falls in the Northwest Fynbos Bioregion of South Africa, a winter-rainfall zone [48] (Fig 1). It is situated on a rock platform some 70 meters above its surroundings, with large rocks, originating from an almost completely eroded peak, resting on the edge of the platform. One of these rocks is shaped like a small pyramid (Fig 2). Inside is a hollow area of about 30 square metres with a maximum height of nearly 2 metres and several concave depressions forming openings to the shelter [24], [25], [39–42].

The site was discovered in 1991 during a rock art survey [40]. In 1993, an occupation layer with a maximum thickness of about 35 cm was excavated from at least two thirds of the floor surface [40], [41]. Larsson [42] conducted a second excavation in 2008. Thus, in contrast to most recent excavations, which cover limited areas of Middle Stone Age sites only, a major part of the occupation layer at Hollow Rock Shelter was excavated.

A recent analysis of the chronology, stratigraphy and spatial distribution of artefacts at the site demonstrated that the Hollow Rock Shelter deposits are almost exclusively associated with a Still Bay lithic industry [39]. The assemblage includes a large number of Still Bay points that were all produced on local raw material or material available at a short distance from the site such as quartzite, quartz and silcrete. Hornfels is also present in the assemblage, for example in the shape of blades, but it was not used for Still Bay point production at the site. There is no evidence of substantial activity at the site during a younger phase, and no evidence of activities older than the Still Bay phase [39]. Optically stimulated luminescence (OSL) analyses from the main artefact-bearing levels provided an age estimate of 80–72 ka [25], [26], [39].

Umhlatuzana Rock Shelter is located about 35 km west of Durban in KwaZulu-Natal in what is currently the Sub-Escarpment Savanna Bioregion, a summer-rainfall zone [48] (Fig 1). It is a north-facing shelter situated on a steep cliff approximately 100 metres above the Umhlatuzana River, and 531 metres above sea level. It is roughly 45 metres long, 6.5 metres deep and has a maximum roof height of about 17 metres [43] (Fig 2). Kaplan [44], [45] excavated six one-metre squares in 28 arbitrary levels as a rescue project in 1985. Four squares reached bedrock at a depth of about 2.6 metres [45].

ML and colleagues re-opened the site in 2006. This exercise provided new OSL age estimates for three Middle Stone Age phases including the late Middle Stone Age, Howiesons Poort and pre-Howiesons Poort/Still Bay, and addressed previous concerns about the integrity of these assemblages [27]. The upper-most level that can be associated with the pre-Howiesons Poort or Still Bay phase, level 25, has an OSL age estimate of 70.5±4.7 [27]. No Still Bay-type or serrated points occur in levels above this dated context. Level 25, however, still contains many Howiesons Poort-like pieces, but the frequency of these diminishes in the subsequent, older levels [45]. We therefore consider the points from levels 26 and 27 to be most representative of the Still Bay phase at the site, and that they have an age of >70 ka. These layers also contain the most formal points that can be ascribed to the Still Bay, including serrated points produced on locally available raw materials such as quartzite, quartz and hornfels [27], [45]. For this study, we have thus chosen to study the production phases of the Still Bay and serrated points from levels 26 and 27. With regards to serrated points, previous morphometric and statistical work by Lombard and colleagues [27] established that they form part of the Still Bay phase at the site, and that they probably represent a regional or local stylistic expression of the technocomplex.

Formal tools other than points are few from Hollow Rock Shelter and layer 26 and 27 from Umhlatuzana Rock Shelter [40], [41], [44], [45], and not produced in the same way as the points. An exception is a few denticulated blades from Umhlatuzana Rock Shelter. Even though initial observations indicate that these were produced in a different way, compared to the points we have studied, we can not exclude that they could have been produced using similar strategies as described for points below. However, these blades are only worked along the edges and do not show surface-covering flaking. They are therefore easy to recognize and separate from point-production strategies we discussed here.

The OSL results from Hollow Rock Shelter and Umhlatuzana Rock Shelter are consistent with age estimates for Still Bay expressions at other sites such as Blombos Cave [28], [29], Sibudu Cave [30], and Apollo 11 Rock Shelter [31]. They are also in line with the revised age estimates of the Still Bay at Diepkloof Rock Shelter [32] (but see [28], [29]). Although no specific analyses of site function have been presented, both sites are interpreted as recurrently used living sites where a range of day-to-day activities took place. Hence, although our focus is on stone tool production, we do not suggest that site function at either locality was limited to knapping.

## Our samples and approach

### Samples

**Fig 3:**
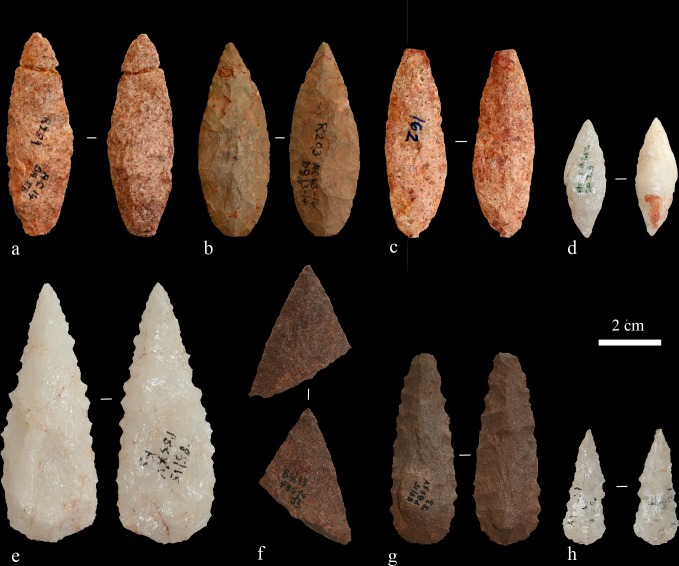
A selection of points from Hollow Rock Shelter (a-d) and Umhlatuzana Rock Shelter (e-h). a) Two glued together pieces of a finished quartzite point, AC14, SAIB, R209. b) A whole finished silcrete point, AC 13–14 and AD 13–14, R203. c) A whole finished quartzite point with broken tip, AE14, 0.854, I162. d) A quartz preform for a point, AC16, IA, R262. e) A whole finished serrated quartz point. f) A quartzite point preform broken during production. g) A finished serrated quartzite point with broken tip. h) A whole finished serrated crystal quartz point. Note that points from Umhlatuzana do not have individual accession numbers.

Previously, AH reported on 69 Still Bay points from Hollow Rock Shelter [25], [39]. This number includes whole and broken points (Fig 3A–3D), as well as preforms and unfinished points that represent different production phases. All points reported by Högberg and Larsson [25] are included in the present study, but now interpreted according to our revised approach as discussed below. The material from Umhlatuzana Rock Shelter comprises Still Bay and serrated points, unfinished points, point preforms and point fragments (Fig 3E–3H). These artefacts were included in the original Kaplan reports [44], [45], announced as part of the Still Bay phase by Lombard and colleagues [27] and have been studied for their morphometric traits by Mohapi [43] (also see [46]). Recently, we also published an analysis of pressure-flaking techniques used to produce some of the Umhlatuzana Rock Shelter points [47].

Kaplan ([45], Table 3) reported a total of 102 unifacial, bifacial and denticulated/serrated points from Umhlatuzana Rock Shelter levels 26 and 27. Lombard and colleagues [27] built their study on the points presented by Kaplan [45], but excluded broken points from their analysis. Mohapi ([43], Table 2) also reports on 64 whole Still Bay and serrated points from levels 25, 26 and 27. In our study, we have analysed 97 points, unfinished points, point preforms and point fragments from levels 26 and 27. The numbers of points in our study and in Kaplan’s vary because our definition of a point differs from his. We have only included points produced with surface flaking, omitting, for example, blades formed to a point by marginal retouch, but we included pieces not previously identified as points and point fragments. We identified most of these additional pieces while working systematically through the core and waste categories (i.e., chips, chunks and flakes) of layers 26 and 27.

The lithic assemblage from Hollow Rock Shelter is held by the Department of Archaeology, University of Cape Town. The lithic assemblage from Umhlatuzana Rock Shelter is held by the KwaZulu-Natal Museum in Pietermaritzburg. The artefacts from Umhlatuzana Rock Shelter do not have individual accession numbers. They are identified in the collection by type, layer (layer 26 and 27) and excavated unit (RBS XXII; PBS XIV; RBS XXIII; PBS XV).

### Approach

The lithic type fossil for the Still Bay was originally defined as a bifacially worked, foliate or lanceolate point with either a semi-circular or wide-angled pointed butt [49]. They are generally described as thin (~10 mm in thickness), with invasive retouch and lenticular cross-sections. Points with unifacial retouch are also present, but less frequent than bifacial points in Still Bay assemblages [15], [49–52] (but see [31]). Although bifacially retouched points are also present in non-Still Bay assemblages, for example, hollow-based points from the final Middle Stone Age at Sibudu Cave and Umhlatuzana Rock Shelter [46], or small quartz points from the Howiesons Poort at Sibudu [53], they generally lack the thin, slender leaf-shaped morphologies characteristic of Still Bay point variations. Their respective contexts are also associated with other trends in lithic production, whereas the production of thin, bifacially knapped points is a key component of the Still Bay technocomplex [1]. Knapping such artefacts involves the understanding of a range of point-thinning techniques and methods (see [54] for general discussion on bifacial technologies, and [15], [25], [55] for Still Bay points). It thus follows that understanding the underlying knapping strategies associated with Still Bay point production, might reveal the transfer of such techno-behavioural knowledge systems across space and/or through time.

Villa and colleagues [15] described the manufacture of Still Bay points as a progressive process, within which clearly distinct production stages might be difficult to define. With this in mind, they chose to use the term ‘production phases’ instead of stages, and divided the production sequence into four phases (phases 1–4), with a subdivision of phase 2 into sub-phases 2a and 2b [15]. This division follows a generally established description of idealised bifacial knapping stages ([56], Figure 8.21). Högberg and Larsson [25] further developed Villa and colleagues’ [15] production sequence, and added an additional sub-phase between 2a and 2b, termed ‘2ab’ ([25], Table 5; Figure 7).

Building on the latter sequence ([25], Table 5), we now propose a five-phase production sequence with refined definitions for each phase of Still Bay point production as observed in the Hollow Rock Shelter and Umhlatuzana Rock Shelter assemblages (see Table 1; note that we exclude the reworked/recycled point phase included in the previous descriptions [15], [25] because it is not a production phase, but part of a tool’s lifecycle). The proposed sequence is in line with what has been described for the similarly thin, leaf-like point-production ‘stages’ for the European Solutrean [57]. Hence, the approach is applicable to invasively retouched point-production sequences regardless of context. In Table 1 we therefore present our proposed sequence together with the ‘stages’ suggested by Aubry and colleagues [57] for Solutrean bifacial points. We agree with Villa and colleagues [15] though, that the production of points is better conceived of as potentially continuous ‘phases in a process’ than as clearly separable stages. In fact, Aubry and colleagues [57] also observed that even though the early (testing) phase could have been separated in space-time, the subsequent knapping sequences were conducted in a continuum of simultaneous thinning and shaping.

**Table 1:** Definitions of production phases used in our study.

Phase	Definition
Phase 1 (not included in the production stages of Aubry et al. [57])	*Blank*: consisting of an unmodified or slightly worked flake, a blade or a nodule. (Aubry and colleagues [57] argue that blank selection would produce distinguishable differences in the resulting remains, with early removals from flake/laminar blanks retaining remnants of the dorsal ridges and of the bulbar surface. They did not include it in their ‘staging’ sequence ([57], page 55), but we see it as an integral part of Still Bay point-production strategies, and therefore start with this phase.)
Phase 2 (corresponds to ‘Early’ stage in Aubry et al. [57])	*Initial shaping*: consisting of a worked piece with a distinct shape, clearly showing the intentions of the knapper to produce a point. The worked piece has several negative removal scars on its surface.
Phase 3 (corresponds to ‘Middle’ stage in Aubry et al. [57])	*Preform shaped as a point*: consisting of a shaped piece with several invasive surface-covering negative flake-removal scars. The edges are regular. The preform is larger than finished points from the same contexts, but the proportions between length, thickness and width demonstrate that the preform can be reduced to a finished point, similar to those in the assemblage.
Phase 4 (corresponds to ‘Late’ stage in Aubry et al. [57])	*Advanced shaping*: consisting of a clearly shaped form with well-balanced proportions. The tip and the base are defined. The edges are pronounced and stable. Commonly, several invasive surface-covering negative flake removals reach over the length axis of the point, i.e., the bilateral equilibrium plane, on one or two faces of the point. The piece appears to be a finished point, but lacks the final retouch or serration along the edges and on the tip.
Phase 5 (corresponds to ‘Finished’ stage in Aubry et al. [57])	*Finished point*.

**Fig 4:**
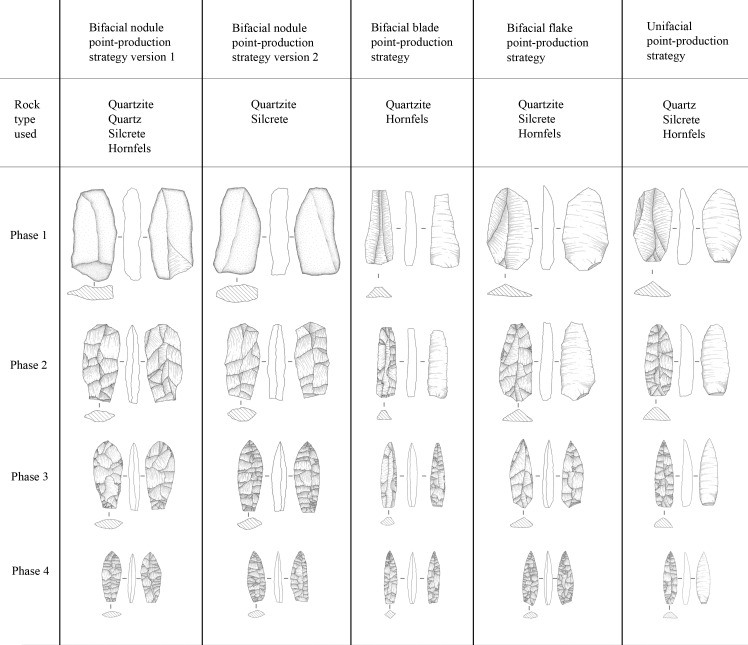
Illustration of the point-production strategies and the first four production phases discussed in the text. Illustrations by Gereth Angelbeck.

Our approach is to elaborate on potential flexibility in Still Bay point-production strategies by describing variability in the production phases as presented in Table 1, as well as in the use of raw materials in point production as represented in the assemblages under investigation. In order to do so, we examined the finished points, unfinished points, point preforms and broken points of the assemblages from Hollow Rock Shelter and Umhlatuzana Rock Shelter. In the discussion presented below, the following constraining factors were taken into consideration (also see [25], page 143, [58], pages 57–59):

1. A phase 1 blank cannot easily be defined in an assemblage, thus even though we know, for example, that quartz nodules were used as blanks, it is not straightforward to conclude that it was brought to the site as blanks;
2. In some instances, not all phases of a point-production strategy can be recognised (Tables 1 & 2, Fig 4);
3. Because of the extensive shaping and invasive retouch that often covers large portions of the surfaces of finished points, such pieces cannot always be confidently assigned to a specific production strategy. The interpretation/identification of a specific production strategy is thus restricted to point-production phases that display diagnostic characteristics of a chosen strategy (see Table 2, Fig 4 and below for detailed outlines of the point-production strategies thus far described for the Still Bay technocomplex).

**Table 2:** Attributes that we used for differentiating point-production strategies, described for a phase 2, 3 and 4 point.

**Cross-section**			
***Point-production strategy***	***Phase 2***	***Phase 3***	***Phase 4***
**Bifacial nodule pps 1**	Lenticular, irregular	Lenticular	Lenticular
**Bifacial nodule pps 2**	Lenticular, irregular	Rhombic, biconvex	Rhombic, biconvex
**Bifacial blade pps**	Wedge-shaped or keeled	Wedge-shaped, keeled or dislocated semi-circular	Diamond shaped
**Bifacial flake pps**	Triangular	Triangular or dislocated semi-circular	Dislocated semi-circular
**Unifacial pps**	Triangular	Triangular or dislocated semi-circular	Semi-circular
**Ridge at the bilateral equilibrium plane on each face of the point**			
***Point-production strategy***	***Phase 2***	***Phase 3***	***Phase 4***
**Bifacial nodule pps 1**	Not clearly defined	Not clearly defined	Centred
**Bifacial nodule pps 2**	Not clearly defined	Off-centred, located towards one of the edges	Off-centred, located towards one of the edges
**Bifacial blade pps**	Follow original ridge on blade on one side. No ridge on the other side	Follow original ridge on blade on one side. Indistinct, not centred or centred on the other	Follow original ridge on blade on one side. Indistinct, not centred or centred on the other side
**Bifacial flake pps**	Follow original ridge on flake on one side. No ridge on the other	Follow original ridge on flake on one side. Indistinct or not centred on the other	Follow original ridge on flake on one side. Indistinct or not centred on the other side
**Unifacial pps**	Follow original ridge on flake or blade on one side. No ridge on the other side	Follow original ridge on flake or blade on one side. No ridge on the other side	Follow original ridge on flake or blade on one side. No ridge on the other side
**Placement of the bifacial equilibrium plane**			
***Point-production strategy***	***Phase 2***	***Phase 3***	***Phase 4***
**Bifacial nodule pps 1**	Centred	Centred	Centred
**Bifacial nodule pps 2**	Not centred	Not centred	Not centred
**Bifacial blade pps**	-	Not centred	Centred
**Bifacial flake pps**	-	Not centred	Not centred
**Unifacial pps**	-	-	-
**Worked on both sides**			
***Point-production strategy***	***Phase 2***	***Phase 3***	***Phase 4***
**Bifacial nodule pps 1**	Yes	Yes	Yes
**Bifacial nodule pps 2**	Yes	Yes	Yes
**Bifacial blade pps**	No	Yes	Yes
**Bifacial flake pps**	No	Yes	Yes
**Unifacial pps**	No	No	No
**Other characteristics**			
***Point-production strategy***	***Phase 2***	***Phase 3***	***Phase 4***
**Bifacial nodule pps 1**			
**Bifacial nodule pps 2**		Away-from-edge knapping using two platforms	
**Bifacial blade pps**		Pressure flaking sometimes used for thinning point	
**Bifacial flake pps**	Platform from original flake visible on point butt	Platform from original flake visible on point butt	Platform from original flake visible on point butt
**Unifacial pps**	Platform from original flake or blade visible on point butt	Platform from original flake or blade visible on point butt	Platform from original flake or blade visible on point butt

**Fig 5:**
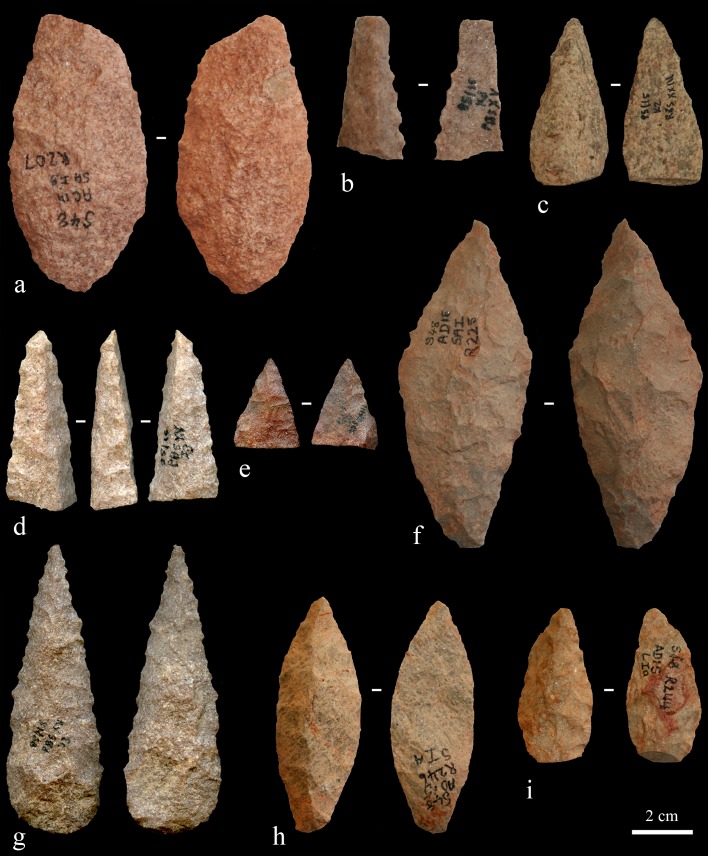
Examples of whole and broken points, as well as unfinished and finished points from the different point-production strategies and in various production phases. a) A bifacial nodule point-production strategy version 1 phase 2 rough-out made of quartzite. b) A bifacial blade point-production strategy phase 2 rough-out made of quartzite. c) A unifacial point-production strategy phase 2 rough-out made of hornfels. d) A bifacial blade point-production strategy phase 3 preform made of quartzite. e) A bifacial nodule point-production strategy version 1 phase 3 preform made of quartzite. f) A bifacial nodule point-production strategy version 2 phase 3 preform made of quartzite. g) A bifacial nodule point-production strategy version 1 phase 5 serrated point made of quartzite. h) A unifacial point-production strategy phase 5 point made of quartzite. i) A bifacial flake point-production strategy phase 4 point made of quartzite. Specimens a, f, h and i are from Hollow Rock Shelter; b-e and g are from Umhlatuzana Rock Shelter. Accession number for the Hollow Rock Shelter artefacts are a: AC14, SAIB, R207; f: AD16, SAI; R225; h: AD15, SIA, R246 and i: AD15, SIA, R244. Points from Umhlatuzana do not have individual accession numbers.

Based on the restrictions highlighted above, the observations and descriptions that follow are largely based on preforms, unfinished points and broken points left in various phases of production (Fig 5). Also, even though it is possible to determine the trend towards the use of a specific point-production strategy in an assemblage, it is not possible to absolutely quantify its presence (see discussion in [25], page 143). Consequently, the numbers and percentages we present below are to be seen as best-fit, qualitative interpretations of observed trends in point-production strategies. With the limitations discussed above in mind, we suggest that our approach will help facilitate comparable spatio-temporal interpretations regarding variability in point-production strategies of Still Bay points, including those that contain serrated and unifacial artefacts, artefacts that are often overlooked in the discussion of the Still Bay technocomplex. This approach does not replace, but complements existing data based on quantitative methods, with a more explicit focus on the tool makers and on variability in reproductive aspects of knowledge-transfer systems [35], [36]. Our aspiration is that future quantitative and/or stylistic approaches will be able to strengthen and/or constrain the interpretations and hypotheses reached by us based on our ‘production strategy’ approach.

## Point-production strategies at Hollow Rock Shelter and Umhlatuzana Rock Shelter

Based on the samples and approach presented above, we have identified five different point-production strategies used for the production of Still Bay points (Fig 4, Table 2). These are the:

- bifacial nodule point-production strategy version 1 (bifacial nodule pps 1);
- bifacial nodule point-production strategy version 2 (bifacial nodule pps 2);
- bifacial blade point-production strategy (bifacial blade pps);
- bifacial flake point-production strategy (bifacial flake pps);
- and unifacial point-production strategy (unifacial pps).

Below we provide full descriptions of our definition of the five point-production strategies, before we present comparative trends between the two assemblages under investigation. The description of the two versions of the bifacial nodule point-production strategy (bifacial nodule pps 1 and bifacial nodule pps 2) and the bifacial flake point-production strategy are modified from Högberg and Larsson [25]. The bifacial blade point-production strategy and the unifacial point-production strategy have not been described previously for Still Bay assemblages.

### Bifacial nodule point-production strategy version 1 (bifacial nodule pps 1)

The knapping starts from a raw, naturally formed or slightly worked nodule or nodule-like flake. From phase 1 to phase 2 the piece is worked into a distinct shape, clearly showing the intentions of the knapper to produce a point (Figs 4 and 5A). Several invasive flakes are detached, using away-from-edge knapping (internal, on-surface percussion). The rough-out is clearly bifacially knapped, with two convex faces on each side of a bifacial equilibrium plane ([59], page 44). From phase 2 to phase 3 the rough-out is worked into a preform shaped as a point. This is achieved through invasive surface-covering flake removals, using away-from-edge as well as on-edge knapping (marginal percussion). Some of the flake removals reach over the length axis of the point, i.e., over the bilateral equilibrium plane ([59], page 44) (Figs 4 and 5E). The edges are kept regular. The preform is large, compared to the finished points, but the proportions between length, thickness and width show the intention of the knapper to reduce the preform to a point. From phase 3 to phase 4 a point with a clear shape and with well-balanced proportions is formed by on-edge knapping. Several invasive surface-covering flakes are detached, reaching over the bilateral equilibrium plane on both faces of the point. The point looks finished, but lacks the final retouch on the edges and the tip. From phase 4 the final retouch and, if pertinent, serration on the edges and the tip, sometimes using pressure flaking [15], [25], [47], result in a phase 5 or finished point (Fig 5G).

The bifacial nodule pps 1 follows the basic concept for typical bifacial shaping ([59], page 44) as has been described, for example, by Whittaker [56] for the production of North American Paleoindian points or by Apel [60], for the production of Late Neolithic Danish daggers. The bifacial nodule pps 1 is recognised by equally well-shaped faces on each side of the bifacial equilibrium plane and its lenticular cross-section from phase 2 to phase 5. Frequently, the bifacial nodule pps 1 shows a centred ridge at the bilateral equilibrium plane on each face of a phase 4 and phase 5 point (Table 2).

### Bifacial nodule point-production strategy version 2 (bifacial nodule pps 2)

**Fig 6:**
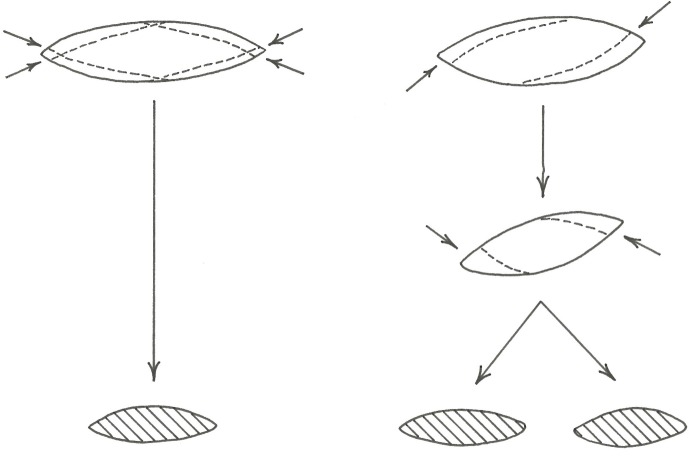
Schematic illustration of the difference in reduction from phases 2 to 4 between bifacial nodule pps 1, left in figure, and bifacial nodule pps 2, right in figure, illustrated with cross-sections. Illustrations by Gereth Angelbeck.

The bifacial nodule point-production strategy version 2 (bifacial nodule pps 2), initially follows the reduction sequence of the bifacial nodule pps 1 (Fig 4). The main difference is in how the reduction is set up, going from phase 2 to phase 4. In the bifacial nodule pps 2, the symmetry of the bifacial rough-out is altered and the edge lines of the biface changed (Fig 6). To knap a phase 2 rough-out into an advanced-shaped phase 4 point according to the bifacial nodule pps 1, the knapper needs to reduce the piece with on-edge knapping from all four platforms (to the left in Fig 6). In this way, the thickness and width is reduced in a manner that controls the lenticular cross-section, the shape of the piece and the centred bifacial equilibrium plane line of the edges. In contrast, using the bifacial nodule pps 2, a phase 2 rough-out is knapped into an advanced-shaped phase 3 preform (Fig 5F) and phase 4 point with away-from-edge knapping. First, the knapper uses two platforms shaping parts of each face, then the two other platforms are used to shape the opposite parts of the faces (to the right in Fig 6).

With the bifacial nodule pps 2, the line of the centred bifacial equilibrium plane is broken, changing the symmetry of the piece and two new edges are created. The lenticular cross-section will change into a rhombic biconvex cross-section, giving rough-outs, preforms and points a slightly twisted look. To stabilise the edges, they are slightly retouched, resulting in a phase 4 point. From phase 4 the final retouch and, if pertinent, serration, on the edges and the tip, sometimes using pressure flaking, results in a phase 5 or finished point.

The bifacial nodule pps 2 is recognised by its twisted look and rhombic biconvex cross-section in phases 3, 4 and 5. The placing of the bifacial equilibrium plane is not centred from phase 2 to phase 5. The bifacial nodule pps 2 shows an off-centred ridge, located towards one of the edges, at the bilateral equilibrium plane on each face of a phase 3, 4 and 5 point (Table 2, Fig 4). Phase 1 and phase 2 from bifacial nodule pps 1 and bifacial nodule pps 2 cannot be distinguished from each other. If the lenticular cross-section is fully recreated in a phase 4 point, it is not possible to tell from that phase 4 point whether it has been knapped with the bifacial nodule pps 1 or bifacial nodule pps 2 (see Fig 6).

### Bifacial blade point-production strategy (bifacial blade pps)

The bifacial blade point-production strategy (bifacial blade pps) does not follow the basic concept for typical bifacial shaping as described by, for example, Inizan and colleagues ([59], page 44). It begins with a blade blank. From phase 1 to phase 2 the rough-out is knapped using the ventral side of the blade as a platform. Invasive flakes are detached to shape the dorsal side (Fig 5B). The majority of these terminate by the ridges on the original blade blank, resulting in a wedge-shaped or keeled cross-section (Fig 4). Occasionally, areas on the dorsal side are left un-flaked, and instead, sections of the lateral edges of the original blade become part of the rough-out outline. Since the ventral side is left unworked, it is not relevant to mention away-from-edge or on-edge knapping in these phases. The knapping performed on the rough-out in this phase is similar to knapping from a plain platform surface. The size of the rough-out is smaller than that used for bifacial nodule pps 1 and bifacial nodule pps 2. Going from phase 2 to phase 3, the rough-out is flipped, and the dorsal side is used as platform to detach invasive flakes running over the ventral side of the original blade. The result is a wedge-shaped, keeled or dislocated semi-circular cross-section. The bifacial equilibrium plane is not centred. The ridge at the bilateral equilibrium plane on each face of a phase 3 preform follow ridges on the original blade on one side and is indistinct, not centred or centred on the other side (Fig 5D). The flipping procedure is repeated going from phase 3 to phase 4. In a recently published study [47], we concluded that pressure flaking was used for thinning some of the Umhlatuzana points, and it seems that this is especially the case for working in phase 3 and phase 4. Throughout the reduction process the outline size of the preform changes less compared to bifacial nodule pps 1 and bifacial nodule pps 2. From phase 4 the final retouch and, if pertinent, serration on the edges and the tip, sometimes using pressure flaking [47], results in a phase 5 or finished point. The bifacial blade pps strategy creates a diamond-shaped cross-section on phase 4 and phase 5 points (Fig 4). Sometimes, the blade is knapped initially using the dorsal side as platform.

The bifacial blade pps is recognised throughout the production phases by the selection of a blade as a blank. In addition, in phase 2 and phase 3 it is recognised by its wedge-shaped or keeled cross-section on rough-outs and preforms. Phase 4 points are recognised by the use of pressure flaking for thinning the point and phase 4 and phase 5 points by their diamond-shaped cross-sections (Table 2, Fig 4). Blades used as blanks are, as far as we can assess, similar to blades reported as part of the Still Bay assemblages at Hollow Rock Shelter [24], and blades present in the Umhlatuzana assemblage [44], [45]. However, we have not specifically analysed blank production in this study. More research is needed to fully understand the characteristics of blanks used in the bifacial blade pps.

### Bifacial flake point-production strategy (bifacial flake pps)

The bifacial flake point-production strategy (bifacial flake pps) is set up in a similar way to the bifacial blade pps. What differs is that the bifacial flake pps starts with a flake blank. Another difference is that we currently have no evidence of the use of pressure flaking for thinning points in phase 3 for the bifacial flake pps, as was the case for the bifacial blade pps. The bifacial flake pps is recognised by its triangular cross-section in phase 2 and phase 3, and its dislocated semi-circular cross-section in phase 4 (Fig 5I). Another trait is that the platform of the original flake blank forms the base of the finished point (Table 2, Fig 4), which has also been noted on Still Bay points from Sibudu Cave [52]. Hypothetically, if a point made with the bifacial flake pps is worked into a lenticular cross-section, and the platform of the original flake blank is removed, then this point-production strategy cannot be distinguished from a phase 4 or phase 5 point made with the bifacial nodule pps 1 or bifacial nodule pps 2.

The flake blank used for the bifacial flake pps is wider than the blade blank used for the bifacial blade pps. It must be straight, and needs to have a long, coherent dorsal ridge running from the platform to the distal end (Fig 4). Based on an analysis of the flake blanks from Hollow Rock Shelter, AH concluded that the flakes necessary for the bifacial flake pps were produced from large cores, each with a facetted platform and straight front with several previous negative removals. Such cores were specialised for producing blanks for this specific pps ([25], page 142), but were not found in the assemblage from the site. Thus, ready-made blanks for this pps were probably brought in to Hollow Rock Shelter.

### Unifacial point-production strategy (unifacial pps)

The unifacial point-production strategy (unifacial pps) starts with a flake or blade blank. From phase 1 to phase 2 the rough-out is knapped using the ventral side of the flake or blade as a platform. Invasive flakes are detached to shape the dorsal side (Fig 5C). The majority of these terminate by the ridge(s) on the original flake or blade blank. In this phase of production, the unifacial pps is comparable to the bifacial blade pps and bifacial flake pps described above, but the approach differs in one important aspect. During the unifacial pps, the ventral side of the flake or blade blank is not worked throughout the whole production process (Table 2, Figs 4 and 5H). This means that from phase 2 to phase 4 the rough-out and preform is formed by invasive flaking, normally covering the whole dorsal side of the original blank. From phase 4 the final retouch and, if pertinent, serration on the edges and the tip, sometimes using pressure flaking [47], results in a phase 5 or finished point with flake scars present only on the dorsal side. The ventral side is left with its original blank surface (Figs 4 and 5H). A few pieces are exceptions, with the flake or blade initially knapped from the dorsal side, and hence with detachment over the ventral side.

This point-production strategy results in unifacially-knapped points, as opposed to the bifacial points most often associated with the Still Bay. Consequently, this pps is recognised first and foremost by its unifacial knapping. Phase 2 rough-outs from the unifacial pps and bifacial blade pps, as well as bifacial flake pps cannot be distinguished from each other. We have not studied blanks used for the unifacial pps and can therefore not specify conditions for blank production or use. It remains to be studied whether blanks for the unifacial pps were produced from the same kind of blanks used for the bifacial blade pps or from cores specialised for the purpose of producing blanks, similar to what has been hypothesised for the bifacial flake pps.

### Observed frequencies of each point-production strategy

As mentioned previously, it is not always possible to pinpoint frequencies for variability in point-production strategies (see discussion in Högberg and Larsson [25], and the pps not known’ column in Table 3). Relevant conclusions can, however, be drawn based on the point-production strategies as observed above. For example:

- The bifacial nodule pps 1 was suggested for the production of Still Bay points at Blombos Cave [15], and Högberg and Larsson [25] reported extensive use of this point-production strategy in the production of Still Bay points from Hollow Rock Shelter (n = 38 or 55.1%). Based on the observations presented here, most of the points (n = 41 or 42%) from levels 26 and 27 at Umhlatuzana Rock Shelter can be assigned to this point-production strategy (Table 3).
- The bifacial nodule pps 2, on the other hand, was recorded to a lesser extent on points from Hollow Rock Shelter (n = 4 or 5.8%), but we have no evidence of this approach in the level 26–27 point assemblage from Umhlatuzana Rock Shelter (Table 3).
- AH has not observed the bifacial blade pps for point production at Hollow Rock Shelter. Here we propose that 29% (n = 28) of the points from levels 26 and 27 at Umhlatuzana Rock Shelter were produced with this pps (Table 3).
- As for the bifacial flake pps, Högberg and Larsson [25] reported a relatively extensive (n = 21 or 30.4%) use of this approach at Hollow Rock Shelter, but at Umhlatuzana Rock Shelter only 9% (n = 9) of the points from layers 26 and 27 seem to be produced in this manner (Table 3).
- The unifacial pps is known to a lesser extent from Hollow Rock Shelter (n = 3 or 4.4%) (reported by Högberg and Larsson ([25], Figure 15) as the unifacial flake *chaîne opératoire*). At Umhlatuzana Rock Shelter, 16.5% (n = 16) of the points from levels 26–27 can be assigned to the unifacial pps (Table 3).

**Table 3:** All points included in our analysis from Hollow Rock Shelter and Umhlatuzana Rock Shelter presented in numbers and percentage of the whole assemblage of points for each point-production strategy (top percentage line) as well in relation to stone type used (bottom percentage line). Note that crystal quartz points from Umhlatuzana Rock Shelter are included in the quartz data below.

Raw material	Bifacial nodule pps 1		Bifacial nodule pps 2		Bifacial blade pps		Bifacial flake pps		Unifacial pps		Pps not known		Totals	
**Hollow Rock Shelter**													**n = 69**	
	n	%	n	%	n	%	n	%	n	%	n	%	n	%
Quartzite	21	30.4	1	1.5	0	0	8	11.6	0	0	2	2.9	32	46.4
		65.6		3.2				25				6.2		100
Silcrete	8	11.6	3	4.3	0	0	13	18.8	3	4.3	1	1.4	28	40.6
		28.6		10.7				46.4		10.7		3.6		100
Quartz	9	13	0	0	0	0	0	0	0	0	0	0	9	13
		100												100
Total	38	55.1	4	5.8	0	0	21	30.4	3	4.35	3	4.35	69	100
**Umhlatuzana Rock Shelter**													**n = 97**	
	n	%	n	%	n	%	n	%	n	%	n	%	n	%
Quartzite	17	17.5	0	0	22	22.7	6	6.2	0	0	3	3	48	49.4
		35.4				45.8		12.5				6.3		100
Hornfels	2	2.1	0	0	6	6.2	3	3.1	15	15.5	0	0	26	26.9
		7.7				23.1		11.5		57.7				100
Quartz	22	22.7	0	0	0	0	0	0	1	1	0	0	23	23.7
		95.7								4.3				100
Total	41	42	0	0	28	29	9	9	16	16.5	3	3.5	97	100
**Point production summary for both sites**													**n = 166**	
	n	%	n	%	n	%	n	%	n	%	n	%	n	%
Quartzite	38	22.9	1	0.6	22	13.3	14	8.4	0	0	5	15.1	80	48.2
		47.5		1.3		27.5		17.5				6.2		100
Quartz	31	18.7	0	0	0	0	0	0	1	0.6	0	0	32	19.3
		96.9								3.1				100
Silcrete	8	4.8	3	1.8	0	0	13	7.8	3	1.8	1	0.6	28	16.9
		28.6		10.7				46.4		10.7		3.6		100
Hornfels	2	1.2	0	0	6	3.6	3	1.8	15	9	0	0	26	15.7
		7.7				23.1		11.5		57.7				100
Total	79	47.6	4	2.4	28	16.9	30	18.1	19	11.4	6	3.6	166	100

The breakdown presented above, and summarised in Table 3, demonstrates both similarities and variations in how point-production strategies were applied at the two sites. We elaborate on these outcomes in the discussion section.

### Point-production strategies in relation to rock type

**Fig 7:**
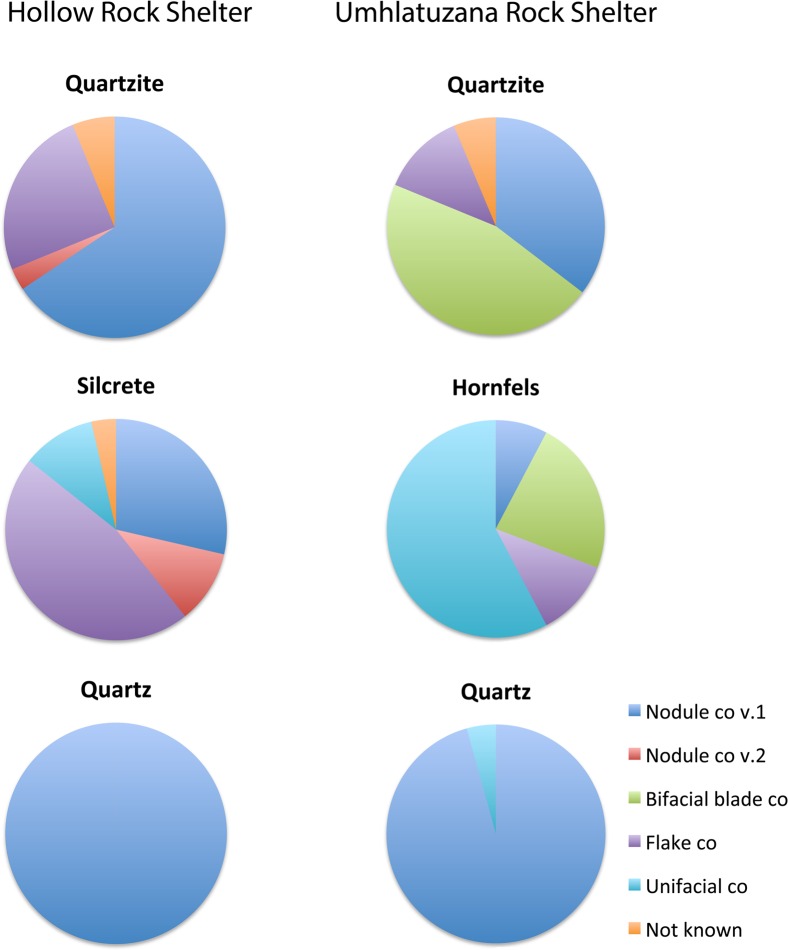
Frequencies of each point-production strategy in relation to rock type for each site. Diagram based on numbers from Table 3.

Further conclusions can be drawn based on variation in point-production strategies as presented above, and the use of rock types for point production at Hollow Rock Shelter and Umhlatuzana Rock Shelter (Table 3, Fig 7). For example, it is clear that at both sites, quartzite was the preferred material for the production of Still Bay points (48.2%), with 46.4% of all the points at Hollow Rock Shelter, and 49.4% at Umhlatuzana Rock Shelter made of this rock type. At both sites, knappers used the bifacial nodule pps 1 to produce quartzite points. At Hollow Rock Shelter, it seems to have been the preferred point-production strategy associated with this rock type, with 65.6% of the quartzite point component produced in this way.

For Umhlatuzana Rock Shelter, we attributed 35.4% of the quartzite points to the bifacial nodule pps 1, which represent the second-most used approach for this material at the site. Here, the bifacial blade pps was the preferred knapping strategy for producing quartzite Still Bay and/or serrated points with 45.8% of all quartzite points manufactured this way. There is no evidence of knappers using this strategy for point production (of any rock type) at Hollow Rock Shelter. Here, however, we found one example of using quartzite for the bifacial nodule pps 2 (Table 3, Fig 7), which according to our observations, was never applied at Umhlatuzana Rock Shelter. At both shelters, knappers also used the bifacial flake pps successfully to produce quartzite points. We have attributed 25% of all quartzite points at Hollow Rock Shelter to this point-production strategy, and 12.5% at Umhlatuzana Rock Shelter. For five quartzite points, we were unable to deduce the point-production strategy with confidence (Table 3).

The availability of rock types used for point knapping in the two different regions where the shelters are located is reflected in the use of silcrete at Hollow Rock Shelter and hornfels at Umhlatuzana Rock Shelter. These rock types were the second-most used for point production during the Still Bay phase at both sites (Table 3). At Hollow Rock Shelter, it seems that the bifacial flake pps was the preferred strategy for the production of silcrete points with 46.4% of all points produced from this material made in this manner. The bifacial nodule pps 1 approach also proved to be successful for the knapping of silcrete points at the site (28.6% of all the silcrete points), with both the bifacial nodule pps 2 and the unifacial pps only represented in less than 11% of all silcrete points each. Using silcrete for the bifacial blade pps was never attempted at the site.

A different pattern emerges for the Umhlatuzana hornfels assemblage. Here, most points made of this rock type seem to have been knapped using the unifacial pps (57.7% of all the hornfels points) (Table 3). The second-most successful approach to knapping points in this material seems to have been the bifacial blade pps represented in 23.1% of the hornfels assemblage. Knappers also used the bifacial flake pps as well as the bifacial nodule pps 1, with the latter being used least often at Umhlatuzana Rock Shelter, and the bifacial nodule pps 2 seemingly never attempted.

At both sites, quartz was used for the production of Still Bay points, but in both cases it was the least used material in this context. At Hollow Rock Shelter, all quartz points were produced with the bifacial nodule pps 1, and at Umhlatuzana Rock Shelter, all but one quartz point was produced in this manner. This might indicate a constraining factor inherent in this rock type that prevented the successful use of other point-production strategies.

In general, the data and interpretations above indicate a marked link between rock type and point-production strategy preferred for the production of points at both sites. An example of this trend relates to the just-mentioned knapping of quartz points. In addition, at both sites the unifacial pps was used only for materials other than quartzite, which might indicate that the properties of quartzite are not conducive to this approach. Future experimental studies are aimed at assessing this inference.

## Thinning flakes as evidence for on-site point production

Högberg and Larsson [25] (also see [39]) presented an analysis of bifacial thinning flakes that provided evidence for the on-site production of Still Bay points at Hollow Rock Shelter. Based on the general knowledge of how typical thinning flakes can be identified (see [23] for a similar approach in identifying such flakes from the assemblage at Sibudu), they defined these as either on-edge thinning flakes with small platforms, or as away-from-edge thinning flakes with somewhat larger platforms. Both thinning-flake types have a platform angle (*angle de chasse*) of 55 ±10 degrees, a diffuse bulb of percussion, a curved shape and two or more negative removals on the dorsal side ([25], Table 6).

**Fig 8:**
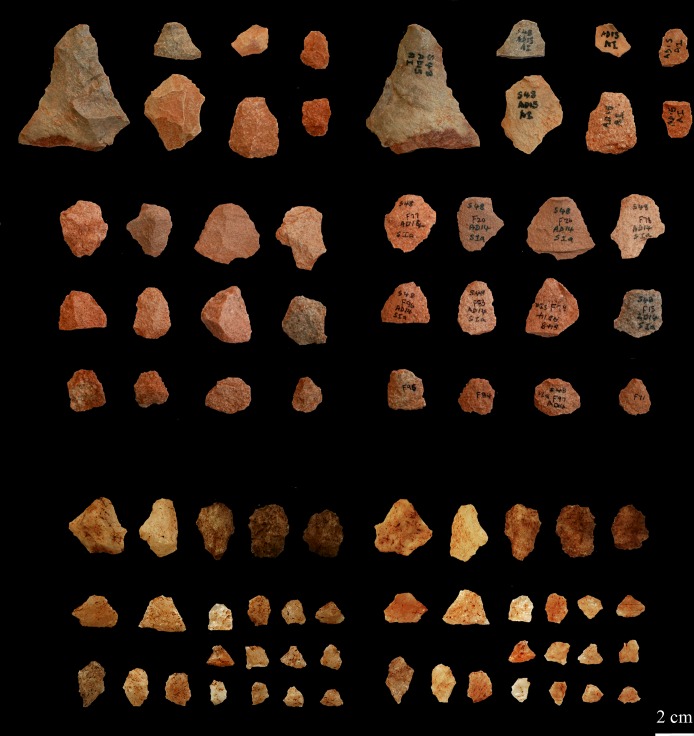
Bifacial thinning flakes of quartzite from Hollow Rock Shelter, dorsal (upper left) and ventral (upper right) sides. Bifacial thinning flakes of quartz from Umhlatuzana Rock Shelter, dorsal (lower left) and ventral sides (lower right).

In the case of the Hollow Rock Shelter assemblage, the presence of large quantities of thinning flakes was interpreted as waste from bifacial nodule pps 1 and bifacial nodule pps 2 (Fig 8) [25]. As shown above, points from Hollow Rock Shelter made of quartzite (n = 32 or 46.4%) and silcrete (n = 28 or 40.6%) are common in the assemblage. Yet, the majority of thinning flakes analysed (n = 204 or 81.9%) are of quartzite. The difference in rock types that the points are made of and the rock types that were knapped on the site (as signified by the thinning flakes), indicates that Still Bay points made from silcrete were sometimes brought to the site. Quartzite and quartz (n = 9 or 13%) Still Bay points, on the other hand, were manufactured on site, most likely from locally available rock types [25]. Interestingly, hornfels was used for blade production, but not for Still Bay point production at Hollow Rock Shelter [24].

Based on the attributes presented by Högberg and Larsson [25], we conducted an analysis of the complete lithic assemblage from layers 26 and 27 from Umhlatuzana Rock Shelter. The purpose was to investigate whether the assemblage contains thinning flakes in quantities that could represent on-site point production. Eight platform attributes referring to size, shape and preparation variables were used [59] (Table 4).

**Table 4:** Attributes used in the analysis of the thinning flakes from the Umhlatuzana Rock Shelter assemblage (modified from Högberg and Larsson [25], Table 6).

Platform variables	Attributes	Definitions used for this analysis
**Size**	Platform width	Distance on platform from one lateral margin to the other in mm ([61], Figure 3.16.1).
	Platform thickness	Maximum distance on platform from dorsal to ventral side in mm ([61], Figure 3.16.1).
**Shape**	Rhombic platform	Flakes with a broken line between the platform and ventral side of the flake (in part equal to hard hammer in Högberg and Larsson [25], Table 6).
	Winged platform	Flakes with a platform that looks like ‘a flying seagull’ in shape ([24], Figure 12).
	Linear platform	Flakes with a smooth line between the platform and the ventral side of the flake (equal to soft hammer in Högberg and Larsson [25] Table 6).
	Punctiform platform	Flakes with a dot-shaped platform.
**Platform preparation**	Abraded	Traces of grinding or rubbing on the edge between platform and the dorsal side on the flake (equals trimmed platform in Högberg and Larsson [25], Table 6).
	Bevelled/ridged	Traces of micro-flaking on the platform or by the platform on the dorsal side of the flake.

Since not all attributes are present on each flake, the total number of registered flakes per attribute differs from attribute to attribute. Högberg and Larsson ([25], Table 6) included the attribute on-edge knapping, defined as flakes with a platform that is 2 mm or less in thickness, and away-from-edge knapping, defined as flakes with a platform that is more than 2 mm in thickness. To obtain a more precise overview of platform size, we measured the actual platform width and thickness. In this study, platforms without abrading and without bevelling represent Högberg and Larsson’s flat platform ([25], Table 6).

**Fig 9:**
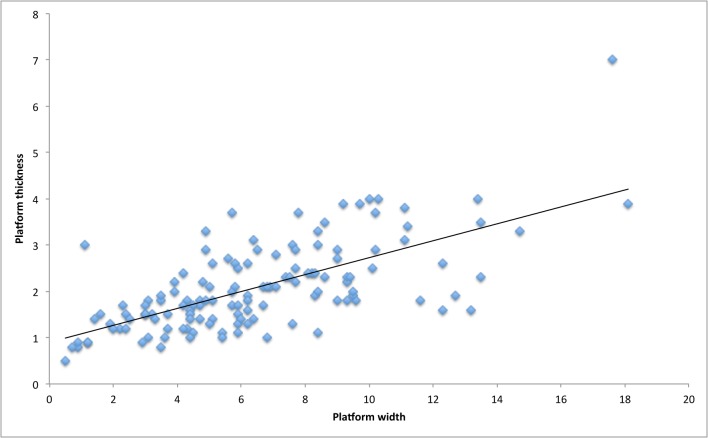
Distribution of platform thickness and width on each bifacial thinning flake from Umhlatuzana Rock Shelter, in mm.

We identified 133 flakes from layers 26 and 27 at Umhlatuzana Rock Shelter as deriving from bifacial knapping (Fig 8). Of these, 76 (57.1%) have platforms that are 2 mm or less in thickness (Fig 9), thus resulting from on-edge knapping. Consequently, platforms on 57 flakes (42.9%) are between 2.1 and 4 mm thick (Fig 9), which is associated with away-from-edge knapping. Whereas platform thickness does not vary much, platform width varies between less than 1 mm up to c. 18 mm (Fig 9).

The most common platform shape for thinning flakes in the Umhlatuzana Rock Shelter assemblage is linear (n = 73 or 54.8%). Winged (n = 38 or 28.6%) and punctiform (n = 22 or 16.5%) platforms cumulatively constitute less than half of the thinning flakes. Ninety-one (68.4%) platforms were abraded. Of these, 59 are linear, 21 are winged and 11 are punctiform. Nineteen flakes have bevelled platforms. Of these, 11 have a lenticular, six a wing-shaped and one a dot-shaped platform. Thirteen flakes have platforms that are both abraded and bevelled. Thirty-six have platforms neither abraded nor bevelled.

The 133 bifacial thinning flakes from layers 26 and 27 from Umhlatuzana Rock Shelter consist of quartz (n = 71 or 53.4%), hornfels (n = 37 or 27.8%) and quartzite (n = 25 or 18.8%) (definition of rock types follow Kaplan [45]). Compared with the rock types recorded for points from the assemblage (Table 3), it is reasonable to conclude that points of all these rock types were knapped on site during the Still Bay phase. However, the differences between the number of points (n = 48) and flakes (n = 25) made of quartzite indicate that points of this rock type might also have been brought into the site. Note that we have followed Kaplan’s [45] identification of hornfels for the flake assemblage. However, during analysis we noticed variability in stone types labelled as hornfels by him. Many of the flakes seemed also to have been made out of mudstone ([43], Table 4). Some of these are weathered to such an extent that it was not possible to analyse them using the attributes described above.

### Pressure flaking flakes

At Hollow Rock Shelter, ephemeral traces of pressure flaking were indicated for a few implements associated with the production of Still Bay points [25]. On the other hand, in our initial analysis of points from Umhlatuzana Rock Shelter, we concluded that pressure flaking was a well-developed knapping strategy at the site used in the production of some Still Bay and serrated points older than 70 ka [47]. We based our interpretation on evidence for the use of push and pull flaking [62], [63]. Patten [62] describes push-flaking *versus* pull-flaking techniques, as resulting in flake scars, and consequently flakes, with different attributes. Flakes produced by pushing tend to be long and slender, and can be considered characteristic of pressure flaking. Flakes resulting from pulling, on the other hand tend to be bulbous with expanding edges [62], and can be achieved through pressure flaking, but are not necessarily indicative of such flaking. These flaking techniques can be combined in numerous ways. According to Patten [62], in their purest forms, push or pull flaking represent ‘extremes in a continuum’. During the process of pressure flaking, however, knappers would apply both push and pull flaking while producing a single artefact.

**Fig 10:**
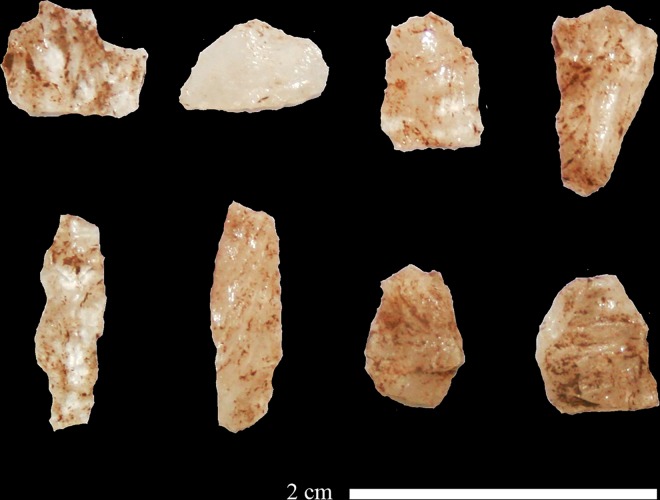
Quartz thinning flakes from Umhlatuzana Rock Shelter; the two to the upper left are pull flakes, and the two to the lower left are push flakes. The four flakes to the right in figure are suggested pull and/or push flakes that cannot be confidently attributed to either category.

During our analysis of the whole assemblage from layers 26 and 27 at Umhlatuzana Rock Shelter, we identified both push and pull flakes (Fig 10). Their presence supports our conclusion that pressure flaking, amongst other techniques, was used to produce Still Bay and serrated points at the site. Apart from serving as indications of pressure flaking, the presence of these flake types further support our interpretation that points were produced at the site.

## Discussion

A key concern of our study is to elaborate on potential variability in knowledge-transfer systems associated with Still Bay point production in southern Africa. Mackay and colleagues [21] concluded that bifacial and unifacial points are present in Still Bay assemblages in different climatic regions (winter-rainfall, year-round and summer-rainfall zones) across southernmost Africa (Fig 1). They emphasise that “it seems unlikely that the appearance of these implements is entirely a consequence of convergence (independent invention in different locations). The more plausible alternative is that the advent of the Still Bay reflects the interaction of populations across southernmost Africa” ([21], page 44). On the other hand, Archer and colleagues ([64], page 58) argue that:

a) Based on “patterns of bifacial point shape and size variation in some key Still Bay assemblages… [m]orphological variation appears to be geographically structured and is driven by the spatial separation between north-eastern and south-western clusters of sites”;

b) “[T]he biogeographic structure of Middle Stone Age populations was complex during the period associated with the Still Bay, and provide little support for heightened levels of cultural interconnectedness between distantly separated groups at this time”.

Thus, in two of the most recent synthetic interpretations we see conflicting explanations for group relations on the landscape based on the analysis of Still Bay points. This dichotomy probably results from different approaches or different theoretical paradigms followed by the respective authors. We suggest that it also reflects the fact that, as a recognised technocomplex in the southern African sequence, the Still Bay has only re-emerged in recent years, after being dropped during the 1960s as a result of vague definition, poor understanding of its place in the Middle Stone Age sequence and its perceived similarity to Solutrean points found in Europe at about 20 ka [2]. During the last two decades, only a handful of Still Bay assemblages have been excavated and dated. Presently, research teams working in southern Africa are thus challenged with unravelling the contexts and conditions of single Still Bay occurrences and sequences, each of these studies contributing to the ever-increasingly rich tapestry of what they might ultimately reveal about the people on the landscape at the time.

Clearly, the two recent hypotheses resulting from regional approaches to demographic aspects of the Still Bay, a) that the Still Bay reflects interaction between populations [21], and b) that the Still Bay provides little support for heightened levels of cultural interconnectedness [64], require further assessment (also see discussion in Soriano et al. [23] on Still Bay assemblages and variability). To do this in relation to our results on variability in point-production strategies between about 80 ka and 70 ka in South Africa, it is necessary to introduce some theoretical perspectives on how to understand past knowledge-transfer systems.

Such socially acquired knowledge systems steered “much of human niche construction” ([65], page 260). The performances of prehistoric tool makers are embedded in the historical contingencies of their socio-cultural contexts and their systems of knowledge transmission [66], [67]. Riede [66] points out that tool-production skills in prehistory would have largely consisted of routine procedures, practised repeatedly from an early age. In the context of hunter-gatherer food-getting strategies teaching and learning include transferring knowledge about ‘hard technologies’ (e.g., how to make a stone point) and ‘soft technologies’ (e.g., the detailed knowledge that foragers use to survive in the world) [68].

A learner who observes a sequence of actions in display [35], for example the production of a stone point, generates a mental image of the sequence. Practicing what has been observed, the learner creates an understanding of ‘what it feels like to execute the action’ ([69], page 2185). This ‘feels like’ includes both the hard and soft aspects of technologies [68]. Advanced intergenerational transmission of knowledge through authentic teaching is unique to humans ([9], [70]). Högberg and colleagues [34] also stress that teaching goes beyond other forms of social learning. It becomes a way to organise society to facilitate the transmission of knowledge. Hence, when we think about knowledge-transfer systems for Still Bay point-production strategies, it is key to understand that the teaching and learning of how to make a point was embedded in day-to-day activities that included knowledge beyond knapping.

Karlin and Julien ([71], page 153) emphasise that the reconstruction of certain tool-production sequences allows archaeologists to discover the processes involved in production technologies, as well as the conceptual patterns from which they originated. The knapper’s work thus reflects society’s knowledge and conventions about how a tool ought to be made. How the knapper applies this knowledge is guided by his/her own intentions or those of the community [72]. This is different from documenting morphometric traits such as shape and size that echo (amongst other things) social norms about style and/or identity (see Bleed [73] and Tostevin [67] for discussion). In this contribution we are, however, concerned with technological knowledge-transfer systems, and suggest that understanding point-production strategies is one way to approach this topic. Such strategies reflect learned and taught principles that can be applied to a variety of pragmatic knapping problems [6], [34], [67]. Of course, stylistic conventions also represent knowledge-transfer systems (see discussion in [67]), which is a topic that we aim to explore in the future to assess and/or augment our current approach.

The transfer of knowledge within or between communities takes different forms. Within a social unit, teaching and learning can be set up in various formal and informal master/apprentice relationships. Active teaching can, for example, take place in various configurations, for example: a) one to one, where the teacher teaches a student; b) one to many, where a group is taught by a teacher; c) many to one, where a person is taught by a group of people; or d) many to many, where individuals teach each other [6], [74]. As opposed to active teaching, learning may also be an individual activity, such as observing and copying without specific instructions (e.g., through play), or take place independently through trial and error [9], [75].

Multifaceted social structures guide how such knowledge-transfer systems are shared within and between groups. Kinship, politics, belief systems, gender and ecology, amongst other things, are examples of such structures [76]. The sharing and exchange of ideas, goods and resources are (re)negotiated and maintained within these structures. Marriage or pair bonding based on kinship is one example of such (re)negotiation, as is migrating groups who introduce new concepts resulting in technological change [77], [78].

Technological change is, however, not straightforward. It involves complex social processes of introduction, negotiation, rejection, acceptance and closure of technologies (the latter implies that a technology has become self-evident) [79]. These processes affect the way new approaches or innovations are accepted or rejected, and determine how existing technologies become merged with or substituted by new ones [80]. For example, a new technology introduced by a migrating group may merge with an existing technology, resulting in an innovation. In the context of socio-economies emerging out of such contact, an innovation may, however, not be accepted by all. In some instances innovations are therefore rejected, and cycles of technological change continues. Once a technological closure is agreed upon, the innovation becomes part of everyday life (see [80] for discussion on technological closure).

An archaeological example of multifaceted knowledge transmission can be seen in blade-production technology during the early Mesolithic in northern Europe. Knowledge was transmitted across long distances through chains of short-distance, non-linear interaction. The result seems to reflect independently developed regional technologies, but they were actually connected through knowledge-transfer systems that reached over large geographical areas [81].

The complex processes presented above, cannot yet be fully explored within the context of the Still Bay. With this study, however, we set out to contribute to the discussion by assessing variability in point-production strategies. In order to do this, we have identified, defined and renamed five different strategies embedded within a 5-phase point-production sequence. We applied this approach to material from two sites with Still Bay assemblages dated to between roughly 80 ka and 70 ka, located in different bioregions and rainfall zones of South Africa (Tables 2 and 3, Fig 4).

One of our outcomes demonstrates a preferred use of the bifacial nodule point-production strategy version 1 (bifacial nodule pps 1) at both Hollow Rock Shelter (55.1%) and Umhlatuzana Rock Shelter (42%). At Hollow Rock Shelter this was the preferred approach used on quartzite (30.4%), and all quartz points at both shelters, bar one at Umhlatuzana Rock Shelter, have been attributed to this approach. Interestingly, bifacial nodule pps 1 is also preferred for Still Bay point production at Blombos Cave [15], [23]. At that site, however, most Still Bay points (71.7%) were made from silcrete, some of which were heat treated [15], [23]. The fact that the bifacial nodule pps 1 approach was used with relative success for all the rock categories represented in point-production at Hollow Rock Shelter and Umhlatuzana Rock Shelter, as well as for the silcrete-dominant Blombos Cave assemblage, indicates a potentially shared convention for Still Bay point-production strategies in South Africa. Hence, on an inter-regional level, there is evidence of a stable technological closure. Society seems to have been organised in a way that facilitated knowledge transmission about conventions of how to make a point, and these shared conventions were applied across regions.

At first glance, our results regarding the preference for the bifacial nodule pps 1 therefore support Mackay and colleagues’ [21] interpretation. It appears that knowledge about this knapping strategy was transmitted over long distances across bioregions and/or rainfall zones. The most parsimonious interpretation would be that such transmission was accomplished through exchange networks amongst neighbouring hunter-gatherer groups of adjacent bioregions, as opposed to long-distance movement of individuals or groups across the landscape. When knowledge-transfer systems are thought of in this manner, it becomes evident that the interpretation of the Still Bay archaeological record does not have to conform to an ‘either-or’ scenario regarding regional interconnectedness, but that key knapping principles for the production of Still Bay points could have been transferred across long distances through chains of short-distance interaction [81].

The two sites under investigation in this study also share the bifacial flake pps, as well as the unifacial approach to Still Bay point production. These conventions could likewise have been shared between groups in the context of an inter-regional technological network facilitated through social processes as described above. What is more, bifacial thinning flakes are present in both assemblages. We therefore conclude that points were knapped at both sites, at least from the blank stages onward. This means that the point-production strategies presented here for each site, were most likely used on-site. Transport to the sites, however, cannot be excluded, most notably that of silcrete points in the context of Hollow Rock Shelter and of some quartzite points in that of Umhlatuzana Rock Shelter. Débitage and point production rejects at Blombos Cave indicate that Still Bay point production was also an important on-site activity there [15].

Regarding site function, it is feasible to suggest that a multitude of day-to-day activities occurred at Hollow Rock Shelter, Umhlatuzana Rock Shelter and Blombos Cave. Good organic preservation at Blombos Cave, where ample faunal and shellfish remains attest to site-related subsistence activities, confirms this interpretation [82]. Organic preservation at both Hollow Rock Shelter and Umhlatuzana Rock Shelter is compromised, but there is no reason to speculate that they were specialised knapping sites, and thus far no spatial analyses have been conducted to establish the possibility of specialised knapping or any other activity areas within these sites [39], [45]. Functional analyses of Still Bay points from Blombos Cave [83] and Sibudu Cave [84] have indicated that some of these artefacts were hafted, and that they were used to tip hunting spears as well as knives. We can therefore assume that they were essential components in day-to-day subsistence and other household activities, apart from any socio-cultural information their possible stylistic traits may have represented.

At closer inspection, our study also reveals fine-grained variations, which we currently interpret as intra-regional developments and transmission of Still Bay point-production strategies. For example, we have demonstrated that quartzite was the preferred material for the production of Still Bay points at both sites. Yet, the knappers at Hollow Rock Shelter mostly used the bifacial nodule pps 1, whereas those at Umhlatuzana Rock Shelter seem to have preferred the bifacial blade pps for the same material.

Another intra-regional or even intra-group variable might be represented in the availability of knappable rocks for Still Bay point production. This variable is represented in the fact that, locally or at short distances from the sites, available silcrete was used at Hollow Rock Shelter and hornfels at Umhlatuzana Rock Shelter. At both shelters, these rocks were used less often than quartzite, but more often than quartz. It would seem that knapping strategies were affected by these materials, because at Hollow Rock Shelter the bifacial flake pps likely was used most often to knap silcrete points, as opposed to the dominant use of the unifacial pps at Umhlatuzana Rock Shelter for the production of hornfels points (Table 3, Fig 7). Thus although the bifacial flake pps and the unifacial pps were used at both sites to manufacture Still Bay points, there seem to be differences regarding their application. This observation might reflect variation in how regional or even local (within group) teaching and learning strategies were applied.

We suggest that currently some of the most convincing evidence for inter-regional variation in Still Bay point production is the fact that two of the point-production strategies here discussed were identified only at one of each of the sites (Table 3). At Hollow Rock Shelter, a limited proportion (5.8% of all points) of the bifacial nodule pps 2 was observed for quartzite (3.2% of all quartzite points) and silcrete (10.7% of all silcrete points). Yet, we have no evidence of its use at Umhlatuzana Rock Shelter. On the other hand, at Umhlatuzana Rock Shelter, knappers often seemed to have used the bifacial blade pps (29% of all points) for the production of quartzite points (45.8% of all quartzite points), and also applied it successfully to hornfels (23.1% of all hornfels points), making it the second-most used point-production strategy used at the site (Table 3, Fig 7). We have found no evidence for the use of this strategy at Hollow Rock Shelter. We therefore suggest that these approaches reflect an intra-regionally developed organisation of knowledge-transfer systems.

Another considerable difference between the two sites is the fact that pressure flaking has been reported only to a minor extent at Hollow Rock Shelter [25]. In contrast, at Umhlatuzana Rock Shelter we have evidence of pressure flaking as an important part of the production technique, often resulting in the uniquely serrated points associated with the Still Bay at the site [27], [47]. In a previous study [47] we found that the Umhlatuzana knappers applied at least three pressure-flaking approaches. These applications seem to include the final shaping of Still Bay points, the deliberate flaking of serrated edges, and the thinning of point preforms. As such, the points from Umhlatuzana Rock Shelter currently represent the most extensive indication of pressure flaking as a well-developed part of the Middle Stone Age knapping repertoire in southern Africa [47]. The extensive use of pressure flaking at this site contrasts with its perceived non-use at Sibudu Cave, located about 90 km from Umhlatuzana Rock Shelter, as reported by Soriano and colleagues [23].

Pressure flaking has also been reported for Blombos Cave [55], but there it seems to be associated with the knapping of heat-treated silcrete. Thus far, no evidence for heat treatment of rocks to improve their knapping properties has been reported for either Hollow Rock Shelter or Umhlatuzana Rock Shelter. Although some experiments demonstrated similar outcomes in the heat treatment of silcrete and quartzite [85], it has been suggested that the process might be useless for most quartzite types [86]. Thus, heat treatment and/or the lack thereof in the production of Still Bay point assemblages might be yet another indicator for inter-regional variability or intra-regional/locally developed and shared knowledge. Future analyses of the spatio-temporal variation in pressure-flaking methods could therefore provide a more in-depth understanding about the complexity of knowledge-transfer systems at work in southern Africa between 80 ka and 70 ka.

Variation in knapping sequences might also be present in the unifacial point component of the Still Bay phase. For example, Mackay and colleagues [21] noticed that large quantities of unifacial points are atypical for Still Bay assemblages from south-western South Africa, but at Apollo 11 Rock Shelter in Namibia [31] unifacial points are common. The relatively frequent application of the unifacial pps (16.5% of all points) at Umhlatuzana Rock Shelter resulting in several unifacial points in layers 26 and 27 is interesting, because it seems more similar to the Apollo 11 Still Bay assemblage in Namibia than to the Cape west coast assemblages. In a future study, we aim to apply the method used in this study to analyse the Apollo 11 Still Bay assemblage to assess this observation.

Based on our current interpretation of similarities and variations recorded in association with our suggested point-production strategies, we propose a blend of knowledge-transfer systems during the Still Bay phase. According to this model, groups shared and adopted some elements of their approaches to point-knapping across southernmost Africa in an inter-regional knowledge-transfer system. Certain knapping conventions, however, were invented or became localised because of intra-regional and/or intra-group knowledge-transfer systems. We are not yet able to explain fully the underlying mechanisms for the observed variability in point-production strategies, such as social dynamics, chronology, demography or palaeoclimatic influences. However, we suggest that both inter- and intra-regional knowledge-transfer systems operated within the context of a flexible approach to Still Bay point knapping, adapted to the needs of a specific group/s and/or individuals in their specific socio-economic and ecological environments [5], [87].

## Conclusion

In this study, we applied a purposely developed approach to discuss variation regarding production strategies for Still Bay points. By focusing on the performances of Still Bay point knappers, we have reintroduced the generative processes into an interpretative framework for thinking about knowledge-transfer systems during the Middle Stone Age of southern Africa [35], [36]. Thus far, our approach points to both similarities and variability amongst assemblages located in two distinct bioregions of South Africa. Based on the outcomes and discussion above, we suggest that knowledge-transfer systems between about 80 ka and 70 ka were complex, and that they indicate a flexible organisation of inter- and intra-regional communication about knapping concepts. Kandel and colleagues [10] concluded that the main signature of the Middle Stone Age is its overall technological variability, which they interpreted as indicating the evolution of behavioural flexibility as a key adaptation. They applied large-scale comparisons to Middle Stone Age technocomplex data that span about 50 thousand years (from about 80 ka to 30 ka) across South Africa. With this study we are, however, able to propose that behavioural flexibility can already be traced within a single technocomplex, namely the Still Bay at the beginning of the phase that Kandel and colleagues [10] investigated. This implies that the development of technological and behavioural flexibility was already in place at the time.
